# Determination of extended-spectrum β-lactamase-producing *Klebsiella pneumoniae* isolated from horses with respiratory manifestation

**DOI:** 10.14202/vetworld.2022.827-833

**Published:** 2022-04-06

**Authors:** Amany A. Arafa, Riham H. Hedia, Sohad M. Dorgham, Eman S. Ibrahim, Magdy A. Bakry, Abeer M. Abdalhamed, Azza S. M. Abuelnaga

**Affiliations:** 1 Department of Microbiology and Immunology, National Research Centre, Dokki, Egypt; 2 Department of Parasitology and Animal Diseases, National Research Centre, Dokki, Egypt

**Keywords:** extended-spectrum β-lactamase, horse, *Klebsiella pneumoniae*, multidrug-resistant, respiratory manifestation

## Abstract

**Background and Aim:**

The World Health Organization considers multidrug-resistant (MDR) *Klebsiella pneumoniae* a major global threat. Horses harbor commensal isolates of this bacterial species and potentially serve as reservoirs for human MDR bacteria. This study investigated antimicrobial resistance in horses caused by extended-spectrum β-lactamase (ESBL)-producing *K. pneumoniae*.

**Materials and Methods:**

One hundred fifty-nine nasal swab samples were collected from horses with respiratory distress not treated with cefotaxime and erythromycin. Biochemical and serological identification was performed on all samples. Polymerase chain reaction (PCR) was used to detect *16S-23S ITS*, mucoviscosity-associated gene *(ma*gA), uridine diphosphate galacturonate 4-epimerase gene *(ug*e), and iron uptake system gene *(kf*u), *bla*_TEM_, *bla*_SHV_, and *bla*_CTX_ genes. Sequence analysis and phylogenetic relatedness of randomly selected *K. pneumoniae* isolates carrying the *bla*_TEM_ gene were performed.

**Results:**

Ten isolates of *Klebsiella* spp. were obtained from 159 samples, with an incidence of 6.28% (10 of 159). Based on biochemical and serological identification, *K. pneumoniae* was detected in 4.4% (7 of 159) of the samples. Using PCR, all tested *K. pneumoniae* isolates (n=7) carried the *16S-23S ITS* gene. By contrast, no isolates carried *mag*A, *uge*, and *kfu* genes. The *bla*_TEM_ gene was detected in all test isolates. Moreover, all isolates did not harbor the *bla*_SHV_ or *bla*_CTX_ gene. Sequence analysis and phylogenetic relatedness reported that the maximum likelihood unrooted tree generated indicated the clustering of the test isolate with the other Gram-negative isolate *bla*_TEM_. Finally, the sequence distance of the *bla*_TEM_ gene of the test isolate (generated by Lasergene) showed an identity range of 98.4-100% with the *bla*_TEM_ gene of the different test isolates.

**Conclusion:**

The misuse of antimicrobials and insufficient veterinary services might help generate a population of ESBL-producing *K. pneumoniae* in equines and humans, representing a public health risk.

## Introduction

Horses are among the most important animals in human history. They are now widely used as sport animals, in wars, for animal-assisted therapy, as a means of transportation, and even for facilitating mining labor. Since then, the frequency of human-domesticated horse contact has progressively increased. Because of the proximity between humans and horses, it is critical to detect infectious illnesses and antimicrobial resistance (AMR) that affect humans and horses [1]. The second most prevalent cause of sickness in horses is a respiratory disorder that can be viral, bacterial, immune-mediated, or mechanical in nature [2].

*Klebsiella* is one of the most common bacteria that can induce serious infections in the respiratory system in humans and animals. Moreover, it can be found on mucosal surfaces, water, food, and soil [3,4]. Virulence factors, such as O-lipopolysaccharide, adherence factors, capsular antigens, and siderophores, contribute to the survival of *Klebsiella* spp. in various environmental conditions. *Klebsiella pneumoniae* is a clinically important member of the genus *Klebsiella*, making it the most significant pathogen of this genus; it can survive high concentrations of disinfectants [5]. The *K. pneumoniae* genome comprises certain virulence genes, such as mucoviscosity-associated gene (*mag*A), uridine diphosphate galacturonate 4-epimerase gene (*uge*), and iron uptake system gene (*kfu*). These genes are responsible for colonization, invasion, and pathogenicity [6]. Most hypermucoviscous *K. pneumoniae* (*hvKp*) isolates belong to the capsular serotypes K1 and K2 [7]. Furthermore, *Klebsiella* is protected from phagocytosis and the bactericidal action of serum by a mucoid capsule [8,9]. Several putative virulence factors, most notably *mag*A and regulator of mucoid phenotype A, have been linked to the hvKp phenotype [10]. The *mag*A gene mediates the hvKp phenotype at first. Further research revealed that *mag*A is responsible for the *K. pneumoniae* capsular serotype K1 [11,12].

*K. pneumoniae* is a multidrug-resistant (MDR) pathogen that represents a growing threat to clinicians worldwide. The frequency of AMR gene determinants, such as extended-spectrum β-lactamase (ESBL), has yet to be determined at the molecular level in Egypt [13]. β-Lactam drugs are the most frequently used antibiotic class for treating infections caused by Enterobacteriaceae, including *Klebsiella* spp. [14]. The progression of the β-lactamase enzyme by *Klebsiella* spp. has spread widely, rendering them resistant to a wide range of antibiotics. Because of indiscriminate antibiotic use, AMR in *Klebsiella-*producing wide-spectrum lactamases, such as ESBL and AmpC lactamases, has evolved, placing the future of β-lactam medications in threat [15,16]. In humans and animals, AMR raises the risk of antimicrobial therapy failure. Furthermore, the emergence of antibiotic-resistant bacteria in companion animals may have public health implications if these bacteria are transmitted to humans [17]. Consequently, determining antibiotic susceptibilities and genetic features of *Klebsiella* spp. that produce ESBL are critical in treating pathogenic infections.

This study aimed to identify *Klebsiella* spp. isolated from horses suffering from respiratory manifestation; evaluate the presence of *mag*A, *kfu*, and *uge* and ESBL-encoding genes (*bla*_CTX-M_, *bla*_TEM_, and *bla*_SHV_); and create a phylogenetic tree to explain the possible genetic link between *bla*_TEM_ gene sequences and other related *bla*_TEM_ gene sequences obtained from GenBank.

## Materials and Methods

### Ethical approval

The study was approved by Medical Research Ethics Committee-NRC (approval no. 19153).

### Study period and location

The study was conducted from February 2020 to January 2021. The study was conducted at National Research Centre, Giza, Egypt. The samples were processed at National Research Centre, Veterinary Research Institute, Microbiology and Immunology Department.

### Sample collection

Nasal swabs were collected from horses with respiratory manifestations from Cairo Governorate, Egypt. Nasal swabs (n=159) were collected from different breeds and ages of horses suffering from respiratory distress and pneumonia, including foreign breed (n=29), native breed (n=73), and Arabic breed (n=57). Moreover, all diseased horses did not take any medication. Nasal swabs were collected using sterile cotton swabs moistened with normal saline from the back of the horse’s nasal cavity (nasopharynx). All samples were transferred to transport media (nutrient broth; Oxoid Ltd., UK). The samples were carefully wrapped, numbered, and sent to the laboratory as quickly as possible within 3-4 h in an icebox.

### Bacterial isolation

The inoculated samples were placed in nutrient broth (Oxoid) tubes and cultured for 20-24 h at 37°C. A loopful of bacteria was spread onto MacConkey agar plates (Oxoid) and incubated aerobically at 37°C for 24-48 h. Suspected colonies (a mucoid and lactose fermenter) were purified by culturing on MacConkey agar (Oxoid) plates. The colony morphology and phenotypic characteristics were evaluated according to Collee *et al*. [18].

### Biochemical identification

Pure colonies were submitted to conventional biochemical assays sucsh as catalase test, oxidase test, oxidative-fermantative test, indol, methyl red, voges-proskauer, citrate test and triple sugar iron agar test [19], and API 20E kits (bioMérieux, Marcy-l’Étoile, France) were used for confirmation. The API 20E findings were 100% according to API web (bioMérieux).

### Serological identification

The Quelling test determined the capsular antigens K1 and K2 in the test isolates [20]. Microscopically, antigen-antibody reactions were seen.

### Determination of the hvKP phenotype

*K. pneumoniae* isolates were isolated from clinical samples, cultured on blood agar medium (Merck, Germany) for 24 h, and incubated at 37°C. Subsequently, the formation of a viscous string of >5 mm in conventional bacteriological loops was used to identify the hvKP phenotype [21].

### Phenotypic detection of ESBL by double-disk synergy test (DDST)

DDST was used to identify ESBL production in confirmed isolates of *K. pneumoniae* [22]. Using a sterile cotton swab, a uniform inoculum was used to inoculate the isolate on Mueller-Hinton agar (equivalent to 0.5 McFarland). On the center of the plate, an Augmentin disk (20 μg amoxicillin and 10 μg clavulanic acid [AMC]) was placed together with test disks of third-generation cephalosporins [30 mg cefotaxime (CTX), 30 mg ceftriaxone (CRO), and 30 mg ceftazidime]. The disks were arranged 20 mm away from the AMC disk (from center to center). The plate was incubated overnight at 37°C. The existence of ESBL-producing *K. pneumoniae* isolates was suggested by an increase in the inhibition zone of any of the four drug disks against AMC.

### Molecular detection of *16S-23S ITS*, ESBL-encoding genes, and certain virulence genes

#### Deoxyribonucleic acid (DNA) extraction

The QIAamp DNA Mini kit (Qiagen, Germany) extracted DNA from samples according to the manufacturer’s instructions, with certain modifications. A 200 µL sample suspension was treated with 10 µL proteinase K and 200 µL lysis buffer for 10 min at 56°C. After incubation, the lysate was given 200 µL of 100% ethanol. The sample was washed and centrifuged according to the manufacturer’s instructions. The nucleic acid was eluted with 100 µL of the kit’s elution buffer.

#### Molecular detection for Klebsiella spp. confirmation gene

Specific oligonucleotide primers (Metabion, Germany) were used for *16S-23S ITS*, forward ATTTGAAGAGGTTGCAAACGAT and reverse TTCACTCTGAAGTTTTCTTGTGTTC, with a molecular weight of 130 bp. All polymerase chain reaction (PCR) mixtures were subjected to 35 cycles of primary denaturation at 94°C for 5 min, secondary denaturation 94°C for 30 s, annealing 55°C for 30 s, extension 72°C for 30 s, and final extension 72°C for 7 min [23].

#### Molecular detection of ESBL-encoding genes

These primers were used for *bla*_TEM_, forward ATCAGCAATAAACCAGC and reverse CCCCGAAGAACGTTTTC, with an amplicon size of 516 bp [24]. PCR for *bla*_SHV_ was done using specific oligonucleotide primers: forward AGGATTGACTGCCTTTTTG and reverse ATTTGCTGATTTCGCTCG, with a molecular weight of 392 bp [25]. The following primers were used for *bla*_CTX_: forward ATGTGCAGYACCAGTAAR GTKATGGC and reverse TGGGTRAARTARG TSACCAGAAYCAGCGG, with an amplicon size of 593 bp. All PCR mixtures for ESBL-encoding genes were subjected to 35 cycles of primary denaturation at 94°C for 5 min, secondary denaturation 94°C for 30 s, annealing 54°C for 40 s, extension 72°C for 45 s (but 40 s for *bla_SHV_*), and final extension 72°C for 10 min [26].

#### Molecular detection of specific virulence genes

Previously published primers were used for *magA* detection at 1282 bp: forward GGTGCTCTTTACATCATTGC and reverse GCAATGGCCATTTGCGTTAG [27]. For *uge*, forward TCTTCACGCCTTCCTTCACT and reverse GATCATCCGGTCTCCCTGTA were used, with a molecular weight of 534 bp [28]. Finally, the *kfu* gene was amplified at 797 bp using primers forward GAAGTGACGCTGTTTCTGGC and reverse TTTCGTGTGGCCAGTGACTC [28].

#### PCR amplification

A 25 µL reaction, including 12.5 µL EmeraldAmp Max PCR Master Mix (Takara, Japan), 1 µL of each primer at 20 pmol concentration, 5.5 µL water, and 5 µL DNA template, was used. A thermal cycler was used to conduct the reaction (Applied Biosystems 2720, USA).

#### Analysis of PCR products

The PCR products were separated through electrophoresis in 1× TBE buffer at room temperature (25°C) using 5 V/cm gradients on a 1.5% agarose gel (Applichem GmbH, Germany). Approximately 15 µL of the products were put into each gel slot for analysis. The fragment sizes were determined using the Gel Pilot 100 and 100 bp Plus DNA ladders (Qiagen). A gel documentation system (Alpha Innotech, Biometra) was used to photograph the gel. Data were evaluated using computer software (Automatic Image Capture Software, Protein Simple formerly Cell Bioscience, USA).

#### Sequence analysis

One isolate of *K. pneumoniae* that carried the *bla*_TEM_ gene was randomly selected for sequence analysis. Each primer was aliquoted into thin-wall PCR tubes at a volume of ~20 µL. The QIAquick PCR product extraction kit was used to purify PCR products (Qiagen, Valencia, California, USA). BigDye Terminator version 3.1 (Applied Biosystems™, USA) cycles sequencing kit was used for the sequence reaction, followed by purification with a Centrisep spin column. The Applied Biosystems 3130 genetic analyzer (Hitachi, Japan) was used to collect DNA sequences. BLAST analysis [29] was used to determine the sequence identity to GenBank accession. A similarity matrix was done using the DNASTAR program (Lasergene version 8.0, https://www.dnastar.com/software/lasergene/) [30]. Phylogenetic analysis was performed with MEGA version 6 using the maximum likelihood approach [31].

## Results

### Isolation and identification of *Klebsiella* spp.

From 159 nasal swabs, 10 *Klebsiella* spp. were recovered from foreign and native breeds with an incidence of 2.5% (4/159) and 3.8% (6/159), respectively. Ten *Klebsiella* spp. were isolated at an incidence of 6.28% (10/159). Finally, *K. pneumoniae* was found at a rate of 4.4% (7/159) based on serological identification.

### Phenotypic detection of ESBL by DDST

Overall, seven ESBL-producing *K. pneumoniae* were detected with an incidence of 42.85% (3/7). CTX and CRO, both third-generation cephalosporins, showed synergism with AMC disk (Augmentin disk). The edge of inhibition produced by CTX and CRO was extended in all three isolates toward the AMC disk.

### Molecular confirmation of *Klebsiella* spp. using *16S-23S ITS*

All *K. pneumoniae* isolates showed positive amplification of the 130 bp fragment.

### Molecular detection of ESBL-encoding genes (*blaTEM*, *bla*_SHV_, and *bla*_CTX_)

Seven *K. pneumoniae* isolates were tested for the presence of ESBL-encoding genes. In Table-1, the *bla*_TEM_ gene was detected in all isolates. Furthermore, *bla*_SHV_ and *bla*_CTX_ were not detected among the test isolates in horses.

**Table 1:** Detection of extended-spectrum β-lactamase encoding genes (*bla*_TEM_, *bla*_SHV_, and *bla*_CTX_), virulence genes (*magA*, *uge*, and *kfu*), and (16S-23S ITS) in *K. pneumoniae* isolates.

*K. pneumoniae* isolate no.	*bla_TEM_*	*bla_SHV_*	*bla_CTX_*	*magA*	*uge*	*kfu*	*16S-23S ITS*
1	+	−	−	−	−	−	+
2	+	−	−	−	−	−	+
3	+	−	−	−	−	−	+
4	+	−	−	−	−	−	+
5	+	−	−	−	−	−	+
6	+	−	−	−	−	−	+
7	+	−	−	−	−	−	+

*K. pneumonia*= *Klebsiella pneumonia*, *mag* A=Mucoviscosity-associated gene, *uge*=Uridine diphosphate galacturonate 4-epimerase gene, *kfu*=Iron uptake system gene

### Molecular detection of *magA*, *uge*, and *kfu* virulence genes

No isolates carried *mag*A, *uge*, and *kfu* from the seven tested isolates, so these isolates were recorded as nonvirulent isolates.

### Sequence analysis and phylogenetic tree

**Figure-1:**
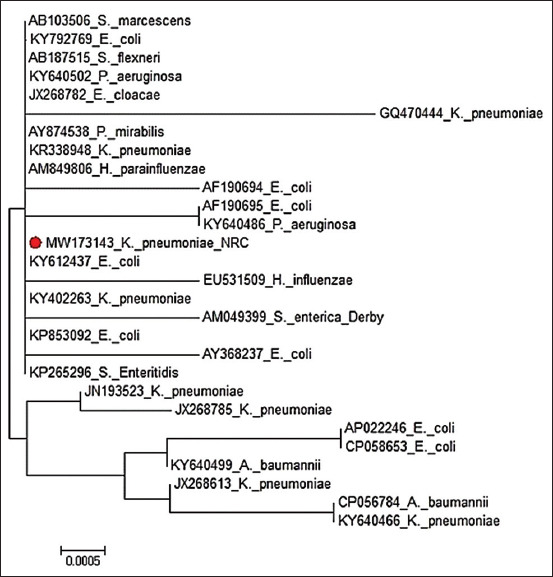
Phylogenetic relatedness of the *bla*_TEM_ gene. The maximum likelihood unrooted tree generated indicates the clustering of the test isolates with the other Gram-negative isolate *bla*_TEM_.

**Figure-2:**
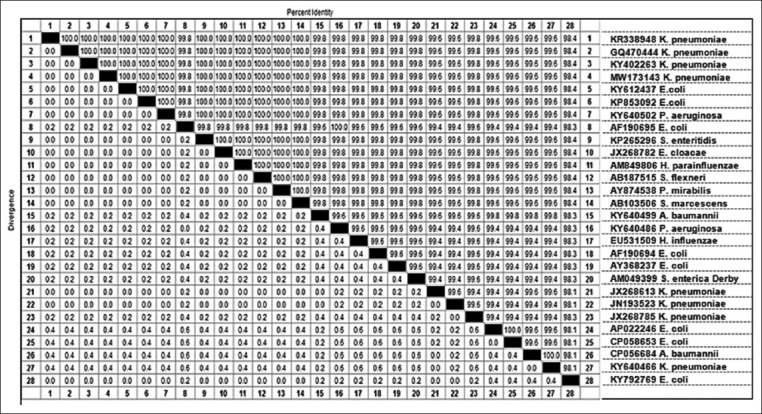
Sequence distance of the *bla*_TEM_ gene of the test isolate (generated by Lasergene) showing an identity range of 98.4-100% with the *bla*_TEM_ gene of different test isolates.

The accession number of the *bla*_TEM_ gene sequence from isolated *K. pneumoniae* was MW173143. Phylogenetic relatedness of the *bla*TEM gene is shown in Figure-1, while the Sequence distance of the *bla*TEM gene of the test strain (generated by Lasergene) is shown in Figure-2.

## Discussion

*Klebsiella* spp. are a prevalent cause of bacterial pneumonia in horses; nevertheless, there are few studies on the clinical presentation and development of the disease [32].

In this study, 10 *Klebsiella* spp. were recovered from foreign and native breeds with an incidence of 2.5% (4/159) and 3.8% (6/159), respectively. Based on serological identification, *K. pneumoniae* was detected in 4.4% (7/159) of the samples. In another study in Egypt, 38/203 samples (74.5%) from 51 horses were positive for bacteria that cause respiratory diseases. *K. pneumoniae* subsp. *pneumoniae* was the most common isolate (26.3%) [33]. This finding was higher than that of Loncaric *et al*. [34], who stated that from 2012 to 2019, *Klebsiella* spp. were isolated from 1541 horses in Austria, yielding 51 (3.3%) specimens that tested positive for *Klebsiella* spp., with an incidence of 3.30%. In addition, four of seven CTX-resistant *Klebsiella* isolates were identified as *K. pneumoniae*. Based on this study, the incidence of *Klebsiella* spp. isolation significantly varies from previous studies; certain factors have been associated with decreased isolation rates, such as different environmental or climatic factors and sanitary management.

The World Health Organization considers MDR *K. pneumoniae* a major global threat. The role of the MDR bacterial population cycling between animals and humans is becoming clearer. Horses harbor commensal isolates of this bacterial species and potentially serve as reservoirs for human MDR bacteria [35].

In horses, *K. pneumoniae* is regarded as a commensal agent, and the clinical significance and severity of sickness are determined by the isolate’s pathogenic potential [36]. The presence of MDR in virulent species of pathogens will significantly impact the health systems and economies of developing countries. *K. pneumoniae* isolates have virulent drug-resistant genes and are resistant to multiple common antibiotics, which is a major worry [37].

This study showed that no isolates carried *magA*, *uge*, and *kfu* genes. By contrast, the *uge* gene was detected at an incidence of 48.6%, distributed in various clinical specimens, such as blood (50%), exudates (48.4%), respiratory secretions (47.3%), and urine (48.8%) [6]. The *uge* gene was found in 84.6% of urinary tract isolates and all blood and respiratory isolates [38]. The occurrence of the *uge* gene in *K. pneumoniae* varied widely (41.6-86%) in different studies [39]. The *kfu* gene is involved in capsule formation and invasiveness and codes for an iron absorption mechanism in 27.8% (103 of 370) of *K. pneumoniae* isolates [40]. The prevalence of the *magA* gene was 62.5% between *K. pneumoniae* isolates [41]. According to the virulence gene results, the previous studies completely contradicted these findings because other virulence genes not included in this study could be carried by the test isolates, which is a reasonable assumption.

MDR ESBL isolates harboring *bla*_CTX-M_, *bla*_OXA_, *bla*_TEM_, *bla*_SHV_, and *bla*_CMY_ have been found in animals, food, and the environment [5]. Phenotypically, based on DDST results, ESBLs were detected at an incidence of 42.85% (three of seven) among all studied *K. pneumoniae* isolates. This finding was supported by Gharrah *et al*. [42], who stated that the incidence of *K. pneumoniae* that produce ESBLs varies by country. Their proportion in Arabian countries is extremely significant (62.5% and 50%). Trigo da Roza *et al*. [34] revealed that *K. pneumoniae* isolated from horses in Portugal were resistant to most antimicrobials tested, including third-generation cephalosporins, fluoroquinolones, and aminoglycosides, and had several AMR genes, including *bla*_ESBL_. In addition, in Shandong Province, China, 14 (6%) isolates of ESBL-producing *K. pneumoniae* were found in 231 environmental samples [43].

Genotypically, this study reported that the *bla*_TEM_ gene was detected in all test isolates. Moreover, all test isolates did not harbor the *bla*_SHV_ or *bla*_CTX_ gene. Abdel-Rhman [44] mentioned that *bla*_SHV_*, bla*_TEM_, and *bla*_CTX-M_ were carried by 68.3%, 56.1%, and 53.7% of *K. pneumoniae* isolates, respectively. Siqueira *et al*. [45] revealed that *bla*_TEM_*, bla*_SHV_, and *bla*_CTX-M_ were detected, and *bla*_TEM_ was nearly found in all ESBL-producing *K. pneumoniae* isolates (5/6 [83.3%]) in horses. Furthermore, Carneiro *et al*. [46] reported that *Klebsiella* isolates were detected in 14.1% of 56 mule foals, primarily ampicillin-resistant, MDR, and ESBL producers. The *bla*_SHV_ gene was more frequently found in *K. pneumoniae* isolates in Brazil.

In Japan, 12 ESBL/AmpC-producing *K. pneumoniae* isolates were identified from seven of 212 (3.3%) healthy Thoroughbred racehorses, indicating that those who work close to racehorses may be at risk of MDR ESBL infections (e.g., veterinarians, caretakers, and owners) [47]. *Klebsiella* spp. isolates were recovered from clinical samples from veterinary clinics in Germany in 2014. For *K. pneumoniae* subsp. *pneumoniae*, the overall ESBL rate was 8%. The majority of *K. pneumoniae* subsp. *pneumoniae* were ESBL producers (29.3%). ESBL genes, such *bla*_CTX-M-1_ (5.6%), *bla*_CTX-M-3_, *bla*_CTX-M-9_, *bla*_SHV-2_, and *bla*_SHV-12_, were also detected [48]. According to a 2018 study in Germany, 1607 *Klebsiella* spp. were recovered from livestock, companion animals, horses, and pets between 2009 and 2016 [49]. The difference between this study and others is the discrepancy between phenotypic and PCR results because these genes are present but rarely expressed.

In this study, sequence analysis and phylogenetic relatedness of the randomly selected isolate carrying the *bla*_TEM_ gene (GenBank accession number MW173143) reported that the maximum likelihood unrooted tree generated indicated the clustering of the test isolate with the other Gram-negative isolate *bla*_TEM_. Finally, the sequence distance of the *bla*_TEM_ gene of the tested strain (generated by Lasergene) showed an identity range of 98.4-100% with the *bla*_TEM_ gene of different test isolates.

This result sounded the alarm that bacteria recovered from horses share a genetic ground with bacteria isolated from other animals and humans [50]. This supported the concept that these animals play a unique role in AMR transfer. Thus, attempts to understand the function of horses better as vectors are critical to public health.

## Conclusion

ESBL-resistant *K. pneumoniae* were confirmed by molecular and phylogenetic analyses for sequenced resistant strains. It is now recognized as a severe public health hazard due to the regular interaction and proximity between horses and humans. More studies are required to understand the spread and virulence of *K. pneumoniae* in horses. The discovery of virulence genes in *K. pneumoniae* strains causing respiratory manifestations that affect horses will aid in the investigation of infectious diseases, help in vaccine development and develop new diagnostic methods for the rapid and accurate identification of changes in *K. pneumoniae*.

## Authors’ Contributions

RHH: Designed the study and critically revised the manuscript. ESI: Collected samples and performed bacterial isolation and biochemical typing. ASMA: Performed serological identification and determination of the hvKP phenotype. SMD: Performed DNA extraction and PCR and drafted, revised, and finalized the manuscript for submission. AMA: Performed the phenotypic detection of ESBL (DDST) and drafted and revised the manuscript. MAB: Interpreted the data and performed phylogenetic analysis. AAA: Designed the study, performed DNA extraction, PCR and sequence analysis, drafted, revised, and finalized the manuscript for submission. All authors have read and approved the final manuscript.

## References

[1] Lönker N.S, Fechner K, Wahed A.A.E (2020). Horses as a crucial part of one health. Vet. Sci.

[2] Arroyo M.G, Slovis N.M, Moore G.E, Taylor S.D (2017). Factors associated with survival in 97 horses with septic pleuropneumonia. J. Vet. Intern. Med.

[3] Nordmann P, Cuzon G, Naas T (2009). The real threat of *Klebsiella pneumoniae* carbapenemase-producing bacteria. Lancet Infect. Dis.

[4] Paterson D.L (2006). Resistance in gram-negative bacteria:Enterobacteriaceae. Am. J. Med.

[5] Wareth G, Neubauer H (2021). The animal-foods-environment interface of *Klebsiella pneumoniae* in Germany:An observational study on pathogenicity, resistance development and the current situation. Vet. Res.

[6] Remya P.A, Shanthi M, Sekar U (2019). Characterisation of virulence genes associated with pathogenicity in *Klebsiella pneumoniae*. Indian J. Med. Microbiol.

[7] Jun J.B (2018). *Klebsiella pneumoniae* liver abscess. Infect. Chemother.

[8] M'lan-Britoh A, Meité S, Boni C, Zaba F, Koffi K.S, Guessennd N, Kakou N.S, Faye-Kette H, Dosso M (2018). First molecular investigation of capsular serotyping and hypervirulent (hvlp) of *K. Pneumoniae* in university hospital center of Yopougon Cote d'ivoire. Afr. J. Clin. Exp. Microbiol.

[9] Ma Y, Bao C, Liu J, Hao X, Cao J, Ye L, Yang J (2018). Microbiological characterisation of *Klebsiella pneumoniae* isolates causing bloodstream infections from five tertiary hospitals in Beijing, China. J. Glob Antimicrob. Resist.

[10] Guo Y, Wang S, Zhan L, Jin Y, Duan J, Hao Z, Lv J, Qi X, Chen L, Kreiswirth B.N, Wang L, Yu F (2017). Microbiological and clinical characteristics of hypermucoviscous *Klebsiella pneumoniae* isolates associated with invasive infections in China. Front. Cell Infect. Microbiol.

[11] Togawa A, Toh H, Onozawa K, Yoshimura M, Tokushige C, Shimono N, Takata T, Tamura K (2015). Influence of the bacterial phenotypes on the clinical manifestations in *Klebsiella pneumoniae* bacteremia patients:A retrospective cohort study. J. Infect. Chemother.

[12] Luo Y, Wang Y, Ye L, Yang J (2014). Molecular epidemiology and virulence factors of pyogenic liver abscess causing *Klebsiella pneumoniae* in China. Clin. Microbiol. Infect.

[13] Khalifa H.O, Soliman A.M, Ahmed A.M, Shimamoto T, Nariya H, Matsumoto T, Shimamoto T (2019). High prevalence of antimicrobial resistance in gram-negative bacteria isolated from clinical settings in Egypt:Recalling for judicious use of conventional antimicrobials in developing nations. Microb. Drug Resist.

[14] Müller-Schulte E, Tuo M.N, Akoua-Koffi C, Schaumburg F, Becker S.L (2020). High prevalence of ESBL-producing *Klebsiella pneumoniae* in clinical samples from central Côte d'Ivoire. Int. J. Infect. Dis.

[15] Rubin J.E, Pitout J.D (2014). Extended-spectrum β-lactamase, carbapenemase and AmpC producing *Enterobacteriaceae* in companion animals. Vet. Microbiol.

[16] Carvalho I, Chenouf N.S, Carvalho J.A, Castro A.P, Silva V, Capita R (2021). Multidrug-resistant *Klebsiella pneumoniae* harboring extended-spectrum β-lactamase encoding genes isolated from human septicemias. PLoS One.

[17] Lee D, Oh J.Y, Sum S, Park H.M (2021). Prevalence and antimicrobial resistance of *Klebsiella* species isolated from clinically ill companion animals. J. Vet. Sci. Mar.

[18] Collee J.G, Marmion B.P, Fraser A.G, Simmons A (1996). Mackie and McCartney Practical Medical Microbiology.

[19] Hansen D.S, Aucken H.M, Abiola T, Podschun R (2004). Recommended test panel for differentiation of *Klebsiella* species on the basis of a trilateral inter-laboratory evaluation of 18 biochemical tests. J. Clin. Microbiol.

[20] Edmondson A.S, Cooke E.M (1979). The production of antisera to the *Klebsiella* capsular antigens. J. Appl. Bacteriol.

[21] Azadpour M, Nowroozi J, Goudarzi G.R, Mahmoudvand H (2015). Presence of qacE_1 and *cepA* genes and susceptibility to a hospital biocide in clinical isolates of *Klebsiella pneumoniae* in Iran. Trop. Biomed.

[22] Elhariri M, Hamza D, Elhelw R, Dorgham S.M (2017). Extended-spectrum beta-lactamase-producing *Pseudomonas aeruginosa* in camel in Egypt:Potential human hazard. Ann. Clin. Microbiol. Antimicrob.

[23] Turton J.F, Perry C, Elgohari S, Hampton C.V (2010). PCR characterization and typing of *Klebsiella pneumoniae* using capsular type-specific, variable number tandem repeat and virulence gene targets. J. Med. Microbiol.

[24] Brisse S, Verhoef J (2001). Phylogenetic diversity of *Klebsiella pneumonia* and *Klebsiella oxytoca* clinical isolates revealed by randomly amplified polymorphic DNA, *gyrA* and *parC* gene sequencing and automated ribotyping. Int. J. Syst. Evol. Microbiol.

[25] Colom K, Pérez J, Alonso R, Fernández-Aranguiz A, Lariño E, Cisterna R (2003). Simple and reliable multiplex PCR assay for detection of blaTEM, blaSHV and blaOXA-1 genes in *Enterobacteriaceae*. FEMS Microbiol. Lett.

[26] Archambault M, Petrov P, Hendriksen R.S, Asseva G, Bangtrakulnonth A, Hasman H, Aarestrup F.M (2006). Molecular characterization and occurrence of extended-spectrum beta-lactamase resistance genes among *Salmonella enterica* serovar Corvallis from Thailand, Bulgaria, and Denmark. Microb. Drug Resist. Fall.

[27] Yeh K.M, Kurup A, Siu L.K, Koh Y.L, Fung C.P, Lin J.C, Chen T.L, Chang F.Y, Koh T.H (2007). Capsular serotype K1 or K2, rather than magA and rmpA, is a major virulence determinant for *Klebsiella pneumonia* liver abscess in Singapore and. Taiwan. J. Clin. Microbiol.

[28] Osman K.M, Hassan H.M, Orabi A, Abdelhafez A.S (2014). Phenotypic, antimicrobial susceptibility profile and virulence factors of *Klebsiella pneumoniae* isolated from buffalo and cow mastitic milk. Pathogen. Glob. Health.

[29] Altschul S.F, Gish W, Miller W, Myers E.W, Lipman D.J (1990). Basic local alignment search tool. J. Mol. Biol.

[30] Thompson J.D, Higgins D.G, Gibson T.J (1994). CLUSTAL W:Improving the sensitivity of progressive multiple sequence alignment through sequence weighting, position-specific gap penalties and weight matrix choice. Nucleic Acids Res.

[31] Tamura K, Stecher G, Peterson D, Filipski A, Kumar S (2013). MEGA6:Molecular evolutionary genetics analysis version 6.0. Mol. Biol. Evol.

[32] Kass P.H, Aleman M (2016). Pneumonia caused by *Klebsiella* spp. in 46 horses. J. Vet. Intern. Med.

[33] Nehal M.F, Osman K.M, Azza N.F, Shaimaa R.A.A, Soumaya S.A.S, Shahein M.A, Ibraheem E.M (2021). Phenotypic study on the bacterial isolates from equine with respiratory disorders regarding antimicrobial drug resistance. World Vet. J.

[34] Loncaric I, Rosel A.C, Szostak M.P, Licka T, Allerberger F, Ruppitsch W, Spergser J (2020). Broad-spectrum cephalosporin-resistant *Klebsiella* spp. Isolated from diseased horses in Austria. Animals.

[35] Trigo da Roza F, Couto N, Carneiro C, Cunha E, Rosa T, Magalhães M, Tavares L, Novais A, Peixe L, Rossen J.W, Lamas L.P, Oliveira M (2019). Commonality of multidrug-resistant *Klebsiella pneumoniae* ST348 isolates in horses and humans in Portugal. Front. Microbiol.

[36] Turton J.F, Baklan H, Siu L.K, Kaufmann M.E, Pitt T.L (2008). Evaluation of a multiplex PCR for detection of serotypes K1.K2 and K5 in *Klebsiella* spp. and comparison of isolates within these serotypes. FEMS Microbiol. Lett.

[37] Fatima S, Liaqat F, Akbar A, Sahfee M, Samad A, Anwar M, Iqbal S, Khan S.A, Sadia H, Makai G, Bahadur A, Naeem W, Khan A (2021). Virulent and multidrug-resistant *Klebsiella pneumoniae* from clinical samples in Balochistan. Int. Wound J.

[38] Aljanaby A.A, Alhasani A.H (2016). Virulence factors and antibiotic susceptibility patterns of multidrug resistance *Klebsiella pneumoniae* isolated from different clinical infections. Afr. J. Microbiol. Res.

[39] Zhang S, Yang G, Ye Q, Wu Q, Zhang J, Huang Y (2018). Phenotypic and genotypic characterization of *Klebsiella pneumoniae* isolated from retail foods in China. Front. Microbiol.

[40] Nahar N, Rashid R.B (2017). Phylogenetic analysis of antibiotic resistance genes and virulence genes of *Klebsiella* species *in silico*. Dhaka Univ. J. Pharm. Sci.

[41] Yu W, Ko W, Cheng K, Lee H.C, Ke D.S, Lee C.C, Fung C.P, Chuang Y.C (2006). Association between *rmp*A and *mag*A genes and clinical syndromes caused by *Klebsiella pneumoniae* in Taiwan. Clin. Infect. Dis.

[42] Gharrah M.M, El-Mahdy A.M, Barwa R.F (2017). Association between virulence factors and extended-spectrum beta-lactamase-producing *Klebsiella pneumoniae* compared to nonproducing isolates. Interdiscip. Perspect. Infect. Dis.

[43] Chi X, Berglund B, Zou H, Zheng B, Borjesson S, Ji X, Ottoson J, Lundborg C.S, Li X, Nilsson L.E (2019). Characterization of clinically relevant isolates of extended-spectrum β-lactamase-producing *Klebsiella pneumoniae* occurring in environmental sources in a rural area of China by using whole-genome sequencing. Front. Microbiol.

[44] Abdel-Rhman S.H (2020). Characterization of β-lactam resistance in *K.pneumoniae* associated with ready-to-eat processed meat in Egypt. PLoS One.

[45] Siqueira A.K, Alves T.S, Franco M.M.J, Ferraz M.M.G, Riboli D.F.M, Paula C.L, Cunha M.L.R, Ribeiro M.G, Leite D.S (2020). Multidrug-Resistant *Klebsiella pneumoniae* Phylogroup KpI in dogs and horses at veterinary teaching hospital. Vet. Med. Public Health J.

[46] Carneiro V.C, Lessa D.A.B, Guttmann P.M, Magalhaes H, Aquino M.H.C, Cunha L.E.R, Arais L.R, Cerqueira A.M.F (2017). Virulence, resistance, and genetic relatedness of *Escherichia coli* and *Klebsiella* spp. isolated from mule foals. Arq. Bras. Med. Vet. Zootec.

[47] Sukmawinata E, Uemura R, Sato W, Thu Htun M, Sueyoshi M (2020). Multidrug-resistant ESBL/AmpC-producing *Klebsiella pneumoniae* isolated from healthy thoroughbred racehorses in Japan. Animals (Basel).

[48] Ewers C, Stamm I, Pfeifer Y, Wieler L.H, Kopp P.A, Schønning K, Prenger-Berninghoff E, Scheufen S, Stolle I, Günther S, Bethe A (2014). Clonal spread of highly successful ST15-CTX-M-15 *Klebsiella*
*pneumoniae* in companion animals and horses. J. Antimicrob. Chemother.

[49] Pulss S, Stolle I, Stamm I, Leidner U, Heydel C, Semmler T, Prenger-Berninghoff E, Ewers C.C (2018). Multispecies and clonal dissemination of OXA-48 Carbapenemase in Enterobacteriaceae from companion animals in Germany, 2009-2016. Front. Microbiol.

[50] Elshafiee E.A, Nader S.M, Dorgham S.M, Hamza D.A (2019). Carbapenem-resistant *Pseudomonas Aeruginosa* originating from farm animals and people in Egypt. J. Vet. Res.

